# Euglycemic diabetic ketoacidosis in association with dapagliflozin use after gastric sleeve surgery in a patient with type II diabetes mellitus

**DOI:** 10.1002/ccr3.2147

**Published:** 2019-04-21

**Authors:** Iouri Banakh, Ross Kung, Sachin Gupta, Kati Matthiesson, Ravindranath Tiruvoipati

**Affiliations:** ^1^ Pharmacy Department Frankston Hospital Frankston Victoria Australia; ^2^ Department of Intensive Care Medicine Frankston Hospital Frankston Victoria Australia; ^3^ School of Public Health, Faculty of Medicine, Nursing and Health Sciences Monash University Frankston Victoria Australia; ^4^ Victorian Diabetes and Endocrine Network Malvern Victoria Australia

**Keywords:** diabetes, electrolyte correction, euglycemic diabetic ketoacidosis, intensive care, sodium–glucose cotransporter 2 inhibitor

## Abstract

Sodium–glucose cotransporter 2 inhibitors (SGLT2Is) can be associated with euglycemic diabetic ketoacidosis (eDKA). Severe metabolic acidosis with extreme electrolyte abnormalities can occur with nonsignificant blood glucose elevations in SGLT2I‐treated patients. Additional risk factors for eDKA include prolonged fasting, major illness, large weight loss, and reductions in insulin doses.

## 1 INTRODUCTION

Sodium–glucose cotransporter 2 inhibitors (SGLT2Is) have become a commonly used class of drugs with the results from the EMPA‐REG OUTCOME study demonstrating reductions in cardiovascular outcomes.^1^ Not only did empagliflozin demonstrate weight loss, HbA1c reduction, and reduction in the need for insulin, but it also has demonstrated benefits in reducing all‐cause mortality, cardiovascular mortality, and hospitalization for heart failure.^1^, ^2^, ^3^ Recently, canagliflozin studies showed similar results to empagliflozin, although the reduction in mortality and cardiovascular outcomes were not as strong.^2^, ^3^ This class of drugs is also associated with reductions in blood pressure and fluid loss.^1^, ^2^, ^3^, ^4^, ^5^, ^6^ Unfortunately, these benefits do come at a cost of increased risk of urinary tract infections, osteoporosis (with canagliflozin), increased risk of fractures, and more recently identified risk of euglycemic diabetic ketoacidosis.^2^, ^3^, ^4^, ^6^ In case of canagliflozin, there was also increased risk of amputations identified from long‐term randomized follow‐up studies, but not large scale observational study.^2^, ^3^, ^4^, ^7^ Euglycemic diabetic ketoacidosis (eDKA) has been reported and is considered to be more frequent in patients with type 1 diabetes when treated with SGLT2Is,^3^, ^8^ however, there have been reports in patients with type 2 diabetes presenting eDKA with various degrees of severity.^9^, ^10^ Here, we report a case of severe DKA due to dapagliflozin with extreme electrolyte abnormalities.

## 2 CASE PRESENTATION

A 64‐year‐old female patient presented to an emergency department with severe shortness of breath and lethargy that was preceded by 3 days of vomiting and reduced oral intake leading to dehydration. She had a recent history of undergoing a gastric sleeve weight loss surgery 4 weeks prior. Her other significant past medical history included hypertension, hypercholesterolemia, gastroesophageal reflux, osteoarthritis, vitamin B12 deficiency, migraines, obesity for which she was treated with the gastric sleeve surgery, in addition to type 2 diabetes mellitus for which she was treated with insulin, metformin, and dapagliflozin. Since she had the surgery she lost 20 kg with insulin dose reductions, while remaining on metformin and dapagliflozin.

On examination, she was noted to be tachypnoeac and tachycardiac with heart rate of 100 beats per minute. Her other physical examination including cardiovascular, respiratory, abdominal, and neurological systems were unremarkable. Arterial blood gas on presentation showed a pH of 6.93 [7.35‐7.45], pO_2_—151 mm Hg [83‐108], pCO_2_ 9 mm Hg [34‐45], HCO_3_ 2 mmol/L [22‐28], lactate 1.5 mmol/L [<2.2], sodium 142 mmol/L [135‐145], potassium 4.3 mmol/L [3.5‐5.0], chloride 123 mmol/L [95‐110], and glucose of 13.5 mmol/L [4.0‐7.8]. Given the modest elevation in glucose, a diagnosis of DKA was not considered at initial presentation, with ketones level not being ordered by the treating physicians. The cause of severe metabolic acidosis was not clear at this stage. She was investigated to exclude ischemic bowel and a computed tomography of her abdomen excluded this.

**Figure 1:**
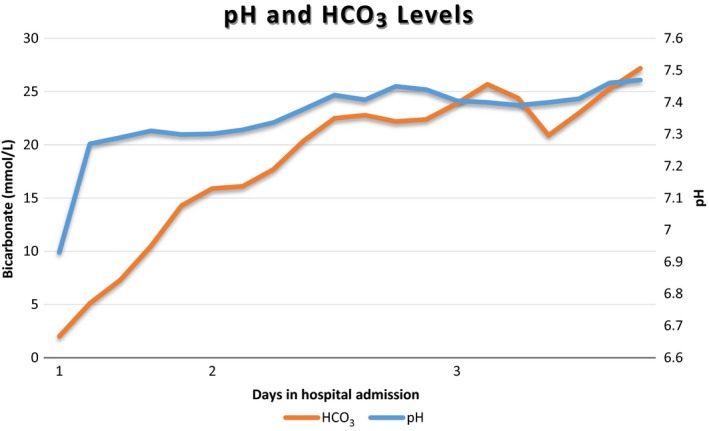
Change in pH and bicarbonate levels during admission

Her treatment included rapid rehydration with 3 L of normal saline administered over 3 hours, along with 10% dextrose and normal insulin. She was also given 300 mL of 8.4% sodium bicarbonate intravenously to correct severe acidosis, leading to improvement in pH (see Figure 1). She was subsequently admitted to the hospital's intensive care unit (ICU) for further electrolyte correction and management of DKA. After 10 hours of hospitalization, in ICU her pathology results had improved with pH of 7.27, blood glucose level (BGL) 9.1 mmol/L, but her ketones remained elevated at 6.9 mmol/L while on an insulin infusion at 2 units per hour with potassium replacement of 60 mmol at the standard rate of 10 mmol/h. After review by an endocrinologist, the diagnosis of euglycemic DKA was established and the rate of insulin and glucose 10% infusion increased to 4 units/h and 80 mL/h, respectively, to resolve ketosis. Twenty‐four hours into patient's treatment, she was still ketotic with level of 3.7 mmol/L with large requirement of potassium replacement and drop in phosphate level to <0.3 mmol/L [0.75‐1.5]. Concurrently, the pH normalized at 7.39 and the patient was planned to be switched to intermediate and short‐acting insulin once oral intake was adequate with cessation of oral hypoglycemic therapy including on discharge. Phosphate was replaced by sodium and potassium phosphate 26.4 mmol infused over 2 hours and regular 1000 mg of oral phosphate tablets administered three times a day. By middle of the second day of admission, patient's ketones fell to 0.4 mmol/L, while still on an insulin infusion at 4 units/h dextrose 10% infused at 80 mL/h. Overnight of the second day, patient BGL dropped to 5.7 mmol/L with insulin infusion being stopped while dextrose 10% continued at 40 mL/h with further 60 mmol of potassium administered to target a level above 4 mmol/L. In the morning of the third day, the ketone level has risen to 2.2 mmol/L and potassium level remained at 3.6 mmol/L. On the fourth day of admission, the patient was transferred to a medical ward with further optimization of her insulin dosing regimen by an endocrinologist with initiation of a combination of intermediate and short‐acting insulin (Novomix 30^®^) at a dose of 6 units twice a day with additional short‐acting insulin dose at of 4 units when BGL were above 12 mmol/L. Her BGL was stable at 11.9 mmol/L, and her phosphate levels improved with oral phosphate dosing dropped down to 1000 mg twice a day, and potassium level at 4.5 mmol/L with oral potassium changed from immediate release 14 mmol tablets to slow release 8 mmol tablets. On the eighth day of admission, the patient was discharged home with Novomix 30^®^ insulin dosed three times a day, 14 units in the morning, 24 units at lunch, and 24 units at night, with an outpatient endocrinology review in the following week.

## 3 DISCUSSION

This case highlights a major issue with use of the new class of oral hypoglycemic in the postoperative period with delayed development and poor recognition eDKA despite the similarity of presenting signs and symptoms to a typical DKA. The mild elevation of BGL during the development of this adverse effect leads to the delayed recognition of the emergency situation and delayed treatment.^11^ The usual BGLs in eDKA is <14 mmol/L, as was the case in our patient, while in a regular DKA it is significantly higher, 26.3 ± 14.8.^11^, ^12^ There are several proposed mechanisms for the development of eDKA including inhibition of insulin secretion through glycosuria, increased glucagon secretion, hypovolemia through reduced sodium reabsorption with increased renal ketone reabsorption.^11^, ^13^ The ketone reabsorption in eDKA leads to a more persistent elevated ketone levels, as was observed during the treatment of the patient described here. SGLT2I action directly on alpha cells in the pancreas may also increase glucagon production and further reduce insulin secretion in patients with type 2 diabetes, contributing further to a ketogenic state with gluconeogenesis and glycogenolysis.^11^, ^14^ Our patient was dehydrated due to prolonged reduced oral intake from severe nausea and vomiting along with reduced insulin doses following the gastric sleeve surgery, exacerbating eDKA and leading to large electrolyte abnormalities with prolonged large dose potassium and phosphate replacement. Unlike a typical DKA with an average pH 7.20 ± 0.02 (IQR 7.09‐7.3), our patient had severe acidosis with a pH of 6.9, requiring a bicarbonate infusion on admission to the hospital.^12^ Her bicarbonate levels were also extremely low at 2 mmol/L and base excess of negative 29 mmol/L, while in typical DKA bicarbonate levels are usually 12.1 ± 6.6 mmol/L and base excess at negative 16 ± 8.^12^ Our patient required 66 hours of ICU treatment and 8 days of hospitalization, which is similar to typical DKA ICU length of stay 41.7 (IQR 24.8‐67.3) hours hospitalization 87 (IQR 50.3‐166) hours.^12^

A differential diagnosis of starvation ketoacidosis for this patient could be considered given the large weight loss within 1 month of admission. However, ketosis and acidosis in starvation ketoacidosis are usually mild compared to eDKA, with reported pH above 7.3 and serum bicarbonate more than 18 mEq/L.^15^, ^16^ Even in prolonged starvation ketoacidosis keto‐anion levels are <5 mmol/L, as circulating free fatty acid (FFA) levels are not high enough to raise the keto‐anion levels higher. In diabetic ketoacidosis, insulin deficiency leads to high serum FFA levels, which in turn increases keto‐anion levels to much higher levels. The presentation pH of our patient was 6.93 and keto‐anion levels after 10 hours of hospitalization were still 6.9 mmol/L, making the diagnosis of starvation ketoacidosis unlikely.

Our patients had several risk factors for the development of eDKA with modest elevation in BGL with insulin dose reductions and prolonged dehydration. Some recommendations identified in the literature to reduce the risk of eDKA in patients on SGLT2I is to avoid SGLT2I use in patients with type 1 diabetes, avoid use during prolonged fasting, dehydration or illness, during major surgery, in patients with excess alcohol intake or with low carbohydrate diets, and avoid reducing insulin doses too fast.^11^

## 4 CONCLUSION

A high index of suspicion is required for patients on dapagliflozin for developing eDKA with modest elevation in BGL especially in patients who have the risk factors such as prolonged fasting associated with dehydration, major surgery, and rapid reduction in insulin dose.

## CONFLICT OF INTEREST

None declared.

## AUTHOR CONTRIBUTION

IB: manuscript formulation, case notes review, and case presentation. RK: preparing case notes and case presentation. SG: manuscript formulation. KM: patient care, reviewing case presentation, and the manuscript. RT: patient care, reviewing case presentation, and formulating the manuscript.
